# Eutrophication influences diversity and community-level change points of mycoplankton in subtropical estuaries

**DOI:** 10.3389/fmicb.2025.1620942

**Published:** 2025-06-27

**Authors:** Jiehao Zhong, Qingxiang Chen, Xiaojie Deng, Yongpeng Guan, Qing He, Rajapakshalage Thashikala Nethmini, Jing Tang, Qinghua Hou, Xiaolei Li, Gonglingxia Jiang, Laizhen Huang, Ke Dong, Nan Li

**Affiliations:** ^1^Key Laboratory of Climate, Resources and Environment in Continental Shelf Sea and Deep Sea of Department of Education of Guangdong Province, Department of Oceanography, Key Laboratory for Coastal Ocean Variation and Disaster Prediction, College of Ocean and Meteorology, Guangdong Ocean University, Zhanjiang, China; ^2^Department of Biological Sciences, Kyonggi University, Suwon-si, Republic of Korea

**Keywords:** mycoplankton, community-level change points, TITAN, subtropical estuary, 18S rRNA gene

## Abstract

Mycoplankton are essential for biogeochemical cycles in natural water bodies. However, the distribution of the mycoplanktonic community and its community-level change points in subtropical estuaries remain unclear. In this study, we employed 18S rRNA high-throughput sequencing to explore the mycoplanktonic community structure and environmental thresholds in the Dafengjiang River Estuary. Agaricostilbomycetes and Saccharomycetes are the dominant classes in the Dafengjiang River Estuary. The alpha and beta diversities of the mycoplanktonic communities showed significant differences (*p* < 0.05) across the seasons. Distance-based redundancy analysis (db-RDA) suggested that the main driver of the total community was eutrophication level, and the key factors for oligotrophication, medium eutrophication, and high eutrophication were dissolved inorganic phosphorus (DIP), ammonium (NH_4_^+^), and chlorophyll-*a* (Chl-*a*), respectively. Threshold Indicator Taxa Analysis (TITAN) exhibited the community-level change points of mycoplankton along the eutrophication gradients were DIP (6–15.5 μg/L), NH_4_^+^ (61.5–62.5 μg/L) and Chl-*a* (2.55–9.3 μg/L), respectively. Random forest analysis revealed that *Rhizophydium*, *Aspergillus* and *Vanrija* were sensitive to eutrophication status and could serve as bioindicator genera for environmental changes. Overall, our study enhances our understanding of the diversity and community-level change points of mycoplankton in subtropical estuaries and lays the theoretical foundation for the environmental monitoring of subtropical estuaries.

## 1 Introduction

Estuaries link the terrestrial and marine environments (Cloern et al., 2016). As a result of anthropogenic activities and climate change, causing widespread eutrophication in coastal areas worldwide (Rabalais et al., 2009). Mycoplankton (also referred to as planktonic fungi) are essential for biogeochemical cycling of natural water bodies (Wang et al., 2012; Grossart et al., 2019; Sen et al., 2022). Recent studies have clearly demonstrated that mycoplankton exhibits significant sensitivity to eutrophication and are highly concerned in the estuarine environment (Wang et al., 2019; Cudowski and Pietryczuk, 2020; Sen et al., 2021; Huang et al., 2022; Lin et al., 2023). For example, mycoplankton communities exhibit reduced diversity and evenness under oligotrophic conditions, whereas the opposite trend is observed under highly eutrophic conditions (Sen et al., 2021). However, the influences of environmental disturbance on mycoplankton distribution remain unclear.

Previous studies have indicated that environmental factors affect the mycoplankton communities in estuaries. For instance, Wang et al. revealed that salinity and trophic status play important roles in shaping the structure of mycoplankton across coastal and estuarine habitats, with high nutrient and low salinity promoting mycoplanktonic abundance (Wang et al., 2019). Huang et al. indicated that salinity, dissolved oxygen, and chemical oxygen demand were the principal environmental parameters governing the structural dynamics of mycoplanktonic communities in the subtropical estuary (Huang et al., 2022). Taylor and Cunliffe (Taylor and Cunliffe, 2016) reported that nitrogen availability is the principal environmental driver of coastal mycoplanktonic community structure in plymouth. Previous research has also suggested that the abundance and diversity of mycoplankton strongly correlate with the trophic status (Wang et al., 2019; Sen et al., 2021), but the effects of eutrophication on mycoplanktonic communities and their thresholds in subtropical estuaries are not fully understood.

The environmental threshold is defined as the critical transition point between two stable states of an ecosystem; once exceeded, the ecosystem swiftly transitions from one state to another (May, 1977; Dala‐Corte et al., 2020). In natural ecosystems, environmental thresholds can be used to analyze abrupt change points where alterations occur in the community (Wu et al., 2023), which are essential for protecting biodiversity and maintaining ecosystem stability (Yang et al., 2017). Currently, classic methods, such as Bayesian analysis and nonparametric changepoint analysis, are used to detect thresholds (Smith and Tran, 2010; Thomsen et al., 2020). Additionally, Threshold Indicator Taxa Analysis (TITAN) is an effective method that is widely employed (Baker and King, 2010; Baker and King, 2013). TITAN can interpret the contributions of taxa on community shifts along environmental gradients (Baker and King, 2013). For instance, Cao et al. (2016) found using the TITAN method that the total nitrogen (TN) thresholds for phytoplankton communities were 1.650 and 1.665 mg/L in a shallow eutrophic Chinese lake. Chen et al. (2023) revealed that zooplankton exhibited distinct temperature protective thresholds between aquatic systems, with riverine thresholds at 19.0°C and lacustrine thresholds at 14.3°C. Huang et al. demonstrated that harmful microalgae display strong environmental adaptability in high-salinity regions and their community structure responds to the threshold of ammonium (57.5–60 μg/L), total phosphorus (27.8–28.5 μg/L), and dissolved inorganic phosphorus (14.5–28 μg/L) along the salinity gradient (Huang et al., 2024). Nevertheless, the community-level change points of mycoplankton in subtropical estuarine ecosystems are poorly understood.

The Dafengjiang River is a subtropical river estuary, serves as primary estuary of Beibu Gulf (Lu et al., 2020). The estuary is characterized by a triangular shape and features a primary channel that discharges into the open Beibu Gulf (Yang et al., 2018). Previous studies have indicated that notable nutrient buildup in the Dafengjiang River Estuary because of human activities, resulting in eutrophication and environmental heterogeneity (Han et al., 2012; Yang et al., 2018). Therefore, to investigate the characteristics of the mycoplanktonic community and its critical transition points within subtropical estuaries, this study applied 18S rRNA high-throughput sequencing to analyze the mycoplanktonic community in seawater samples from the Dafengjiang River Estuary in Beibu Gulf in 2018 and 2020. This study aims to indicate the (a) distribution of mycoplankton, (b) community-level change points of mycoplankton, and (c) important mycoplankton indicator genera in subtropical estuaries. The results of this study provide novel perspectives on the impacts of environmental disturbances on mycoplanktonic communities and maintenance of biodiversity mechanisms, thereby providing initial support for environmental monitoring in subtropical estuaries.

## 2 Materials and methods

### 2.1 Sampling collections and environmental parameters analysis

**Figure 1:**
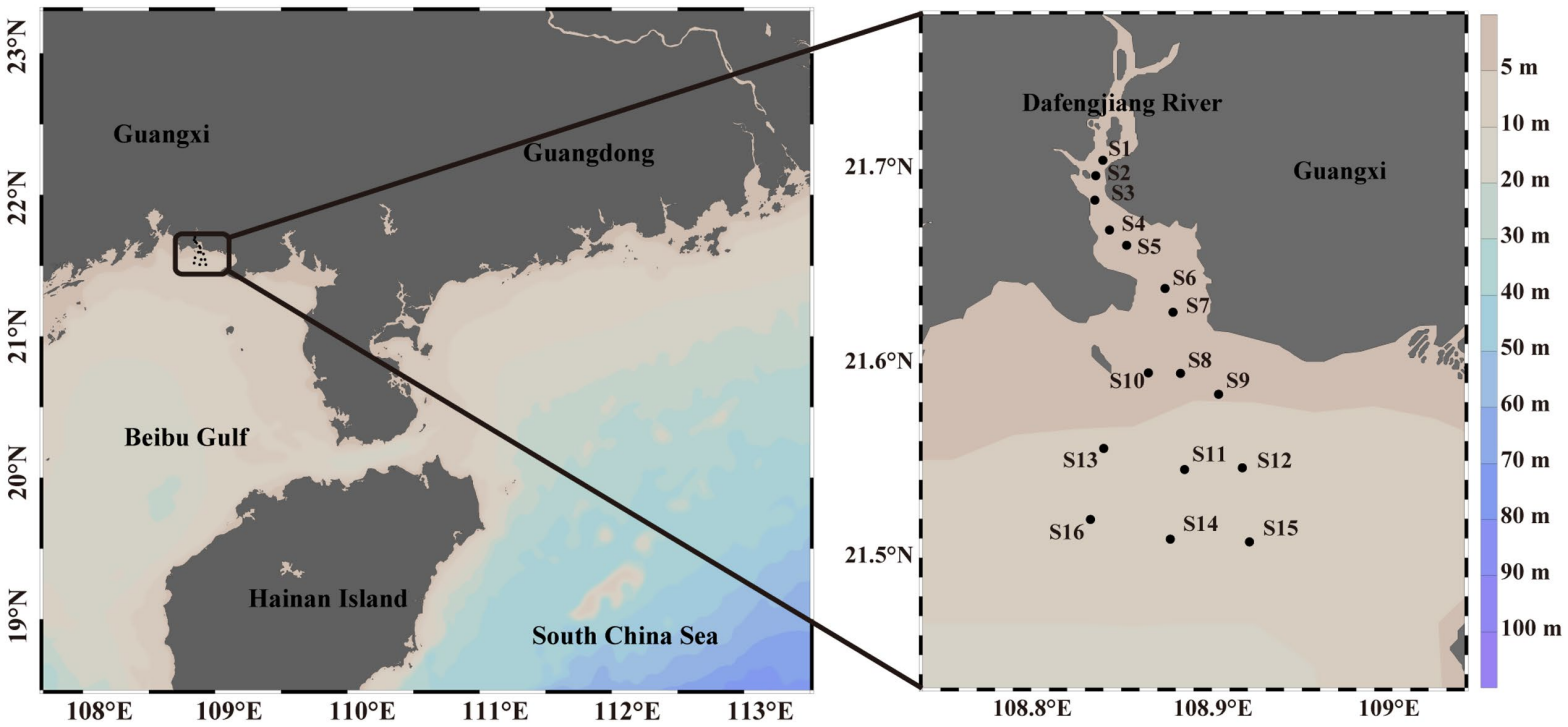
Localization of the sixteen sampling locations in the Dafengjiang River Estuary.

The sampling sites were located in the Dafengjiang River in Beibu Gulf, close to the Guangxi Zhuang Autonomous Region in China (Figure 1). In 2018, surface seawater (0.5 m deep) was sampled quarterly from 15 sites. Similarly, samples were gathered from 16 sites in 2020. Using the five-point sampling method, five water samples were gathered from a square region measuring 5 m × 5 m utilizing 5 L Niskin bottles at each site. In 2018, S8 site in all four seasons and S14 site in spring were unavailable, resulting in 615 samples. According to the distribution of sampling locations, we divided the Dafengjiang River Estuary into three zones: upper reaches (S1-S4), middle estuary (S5-S10) and inner shelf (S11-S16). After being collected, the samples were immediately stored at 4°C and promptly transported to the laboratory.

The environmental parameters of the samples were thoroughly assessed as well (Supplementary Table S1). At each site, readings for temperature, pH, salinity, and dissolved oxygen (DO) content were taken with a portable meter (556 MPS; YSI, USA). The levels of NO_2_^−^, NO_3_^−^, NH_4_^+^, total nitrogen (TN), and total phosphorus (TP) were accurately measured using a flow injection analyzer (Seal-AA3, Germany). Chlorophyll-*a* (Chl-*a*) concentration was measured by a spectrophotometric method (American Public Health Association, 1926). The analysis of total organic carbon (TOC) was conducted using a total organic carbon analyzer (TOC-VCPH, Shimadzu, Japan). Chemical oxygen demand (COD) was measured using alkaline potassium permanganate method. Dissolved inorganic nitrogen (DIN) was determined by the sum of NO_2_^−^, NO_3_^−^, and NH_4_^+^. Base on the concentration of PO_4_^3−^-P to estimate the dissolved inorganic phosphorus (DIP) levels (Lai et al., 2014). The calculation formula for eutrophication index (EI) was calculated as follows: EI = (DIN × DIP × COD × 10^6^)/4,500 (Lai et al., 2014; Chen et al., 2016; Xu et al., 2018). According to Xu et al. (2018), eutrophication can be broken down into three distinct categories: oligotrophication (EI < 1), medium eutrophication (1 < EI < 9), and high eutrophication (EI > 9).

### 2.2 DNA extraction, PCR amplification and high-throughput sequencing

For DNA extraction, 3 L of the seawater samples were filtered using 0.22 μm pore-size polycarbonate membranes (Millipore Corporation, Billerica, MA), followed by kept at −20°C for subsequent analysis. The extraction of environmental DNA from 0.22 μm membranes through a DNeasy PowerWater kit (Qiagen, USA), strictly following the protocol outlined by the manufacturer. The yield and purity of DNA were measured by a NanoDrop 2000 spectrophotometer (Thermo Scientific, USA) and only the samples that passed muster were selected for subsequent experiments. The dual-bar coded primers TAReuk454FWD1 (5′-CCAGCASCYGCTAGTAATTCC-3′) and TAReukREV3 (5′-ACTTTCGTTTGATYRA-3′) were employed to amplify the V4 region (~380 bp) of the 18S rRNA (Logares et al., 2012). The PCR reactions were performed in 20 μL volumes containing 1 × Taq PCR Mastermix (TIANGEN, China), 0.5 μM of each forward and reverse primer, and about 50 ng of template DNA, with the final reaction volume adjusted using double-distilled water. PCR conditions were as follows: 2 min initial denaturation at 98°C, followed by 10 cycles of 10 s denaturation at 98°C, 30 s annealing at 53°C, 30 s elongation at 72°C, followed by 15 cycles of 10 s denaturation at 98°C, 30 s annealing at 48°C, and 30 s elongation at 72°C, with a final extension for 2 min at 72°C. Subsequently, sequencing products were processed using the TruSeq DNA kit (Illumina, USA) per manufacturer specifications.

The Illumina library preparation protocols were followed to prepare the purified library and sequenced on an Illumina MiSeq platform at Majorbio Biotech (Shanghai, China). Using QIIME2, raw sequences underwent processing and quality checking. Sequences with primer mismatches or lengths < 275 bp, low-quality reads (quality scores < 30), and barcode sequences were all removed (Caporaso et al., 2010). The detection and elimination of chimeric sequences were carried out by UCHIME (Edgar et al., 2011). In QIIME2, the prepared reads underwent further dereplication, chimera filtering, and operational taxonomic units (OTUs) were clustered (*de novo*) at 97% similarity (Zhao et al., 2023). OTUs were categorized using the Silva database (Quast et al., 2013), with only fungal sequences were reserved. The sequences are available in GenBank (accession numbers: PRJNA1044415 and PRJNA866330).

### 2.3 Community-level change points analysis

The fungal community responds to environmental factors using TITAN with R package ‘TITAN2’ (Baker and King, 2010). TITAN was applied to determine compositional variations in taxa along environmental gradients, and evaluate the synchronization of taxonomic change points as proof of community thresholds (Baker and King, 2010).

Briefly, the performance of each taxon with distinct pressure variations was evaluated using the IndVal score, including the frequency of occurrence, abundance, and direction of response to changes. The indicator reliability and purity of the threshold were tested using bootstrapping. The final summation of the calculated fraction of each taxon to the environmental pressure determines the fungal community’s responsiveness threshold to the pressure, known as sum-z (Chen et al., 2023). The sum-z was employed to differentiate between the negative (z-) and positive (z+) taxa response to environmental factors, and tracked cumulative responses of declining [(z-)] and increasing [(z+)] taxa in the community. Near this threshold, the abundance of the fungal taxa exhibited the largest aggregate change.

After TITAN identified the initial mutation point of species, the thresholds and the reliability of the relational species were subsequently validated and confirmed based on reliability (reliability ≥ 0.95) and purity (purity ≥ 0.95).

### 2.4 Statistical analysis

The statistical analyses for this study were primarily conducted in R using the ‘ggplot2,’ ‘vegan,’ ‘randomForest,’ and ‘psych’ packages. Alpha diversity was calculated using Shannon’s index (Good, 1953). Beta diversity analysis of the mycoplankton community was conducted using Bray–Curtis distance. Bray–Curtis distance-based redundancy analysis (db-RDA) was used to reveal the community and its connection with environmental parameters. The random forest analysis was utilized to reveal important bioindicators for classification.

## 3 Results

### 3.1 Species composition and diversity of mycoplankton

In total, 4,830,327 fungal sequences were obtained from 615 samples. Subsequently, these fungal sequences were classified into 23,606 OTUs at a 97% similarity threshold. The sample exhibited the Good’s coverage exceeding 92.73%, indicating that the most mycoplanktonic taxa were captured.

**Figure 2:**
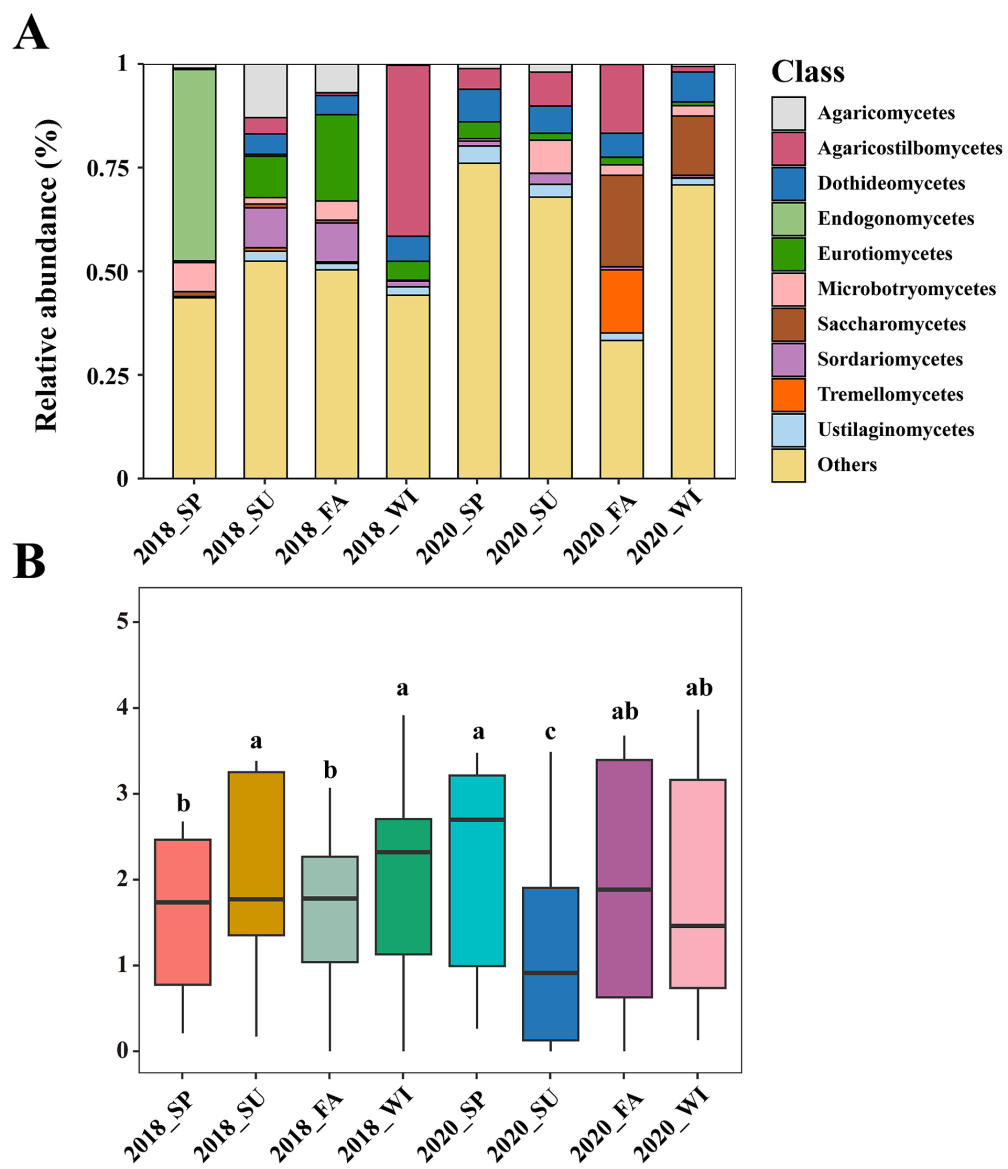
**(A)** Mycoplanktonic community composition at different years (2018 and 2020) and seasons in Dafengjiang River Estuary were evaluated based on relative abundance of different taxa at the class level. **(B)** The alpha diversity (Shannon) at different years (2018 and 2020) and seasons. The alpha diversity shown by boxplot. SP, spring; SU, summer; FA, fall; WI, winter.

At the class level, the top 10 were selected as the most abundant classes present in the mycoplanktonic communities, and the species composition across different samples demonstrated significant variation (Figure 2A). Agaricostilbomycetes (15.58%) and Saccharomycetes (7.90%) dominated the mycoplanktonic community of the Dafengjiang River (Figure 2A). Besides, Endogonomycetes (46.12%) exhibited dominance in the spring of 2018, whereas Agaricostilbomycetes (41.22%) emerged as the most dominant class during the winter of 2018 (Figure 2A). In the taxonomic hierarchy, species that were either relatively rare or could not be definitively recognized were categorized as “Others.” As a result, 51.28% of the mycoplankton at the class level was classified under “Others” (Figure 2A). The dominant class differed at different areas in the Dafengjiang River Estuary during the same season (Supplementary Figure S1). In summary, the mycoplanktonic composition changed in each group, suggesting that the composition of the mycoplanktonic community varied seasonally and regionally.

Alpha diversity in distinct samples was described using Shannon’s indices. A significant difference was observed (*p* < 0.05) in each group across all the samples (Figure 2B). Among the eight groups, the Shannon indices exhibited the highest mean values in spring 2020 and the lowest mean values in summer 2020 (Figure 2B). In addition, there are seasonal differences in the Shannon indices of mycoplankton in different regions (Supplementary Figure S2).

Dissimilarity in mycoplanktonic community compositions was indicated using the Bray–Curtis distance. The results demonstrated that beta diversity of the mycoplanktonic community differed significantly between years and seasons (*p* < 0.05), indicating that the composition of the mycoplanktonic community exhibited distinct spatiotemporal dynamics (Supplementary Figure S3).

### 3.2 Key influencing environmental factors for mycoplanktonic communities

**Figure 3:**
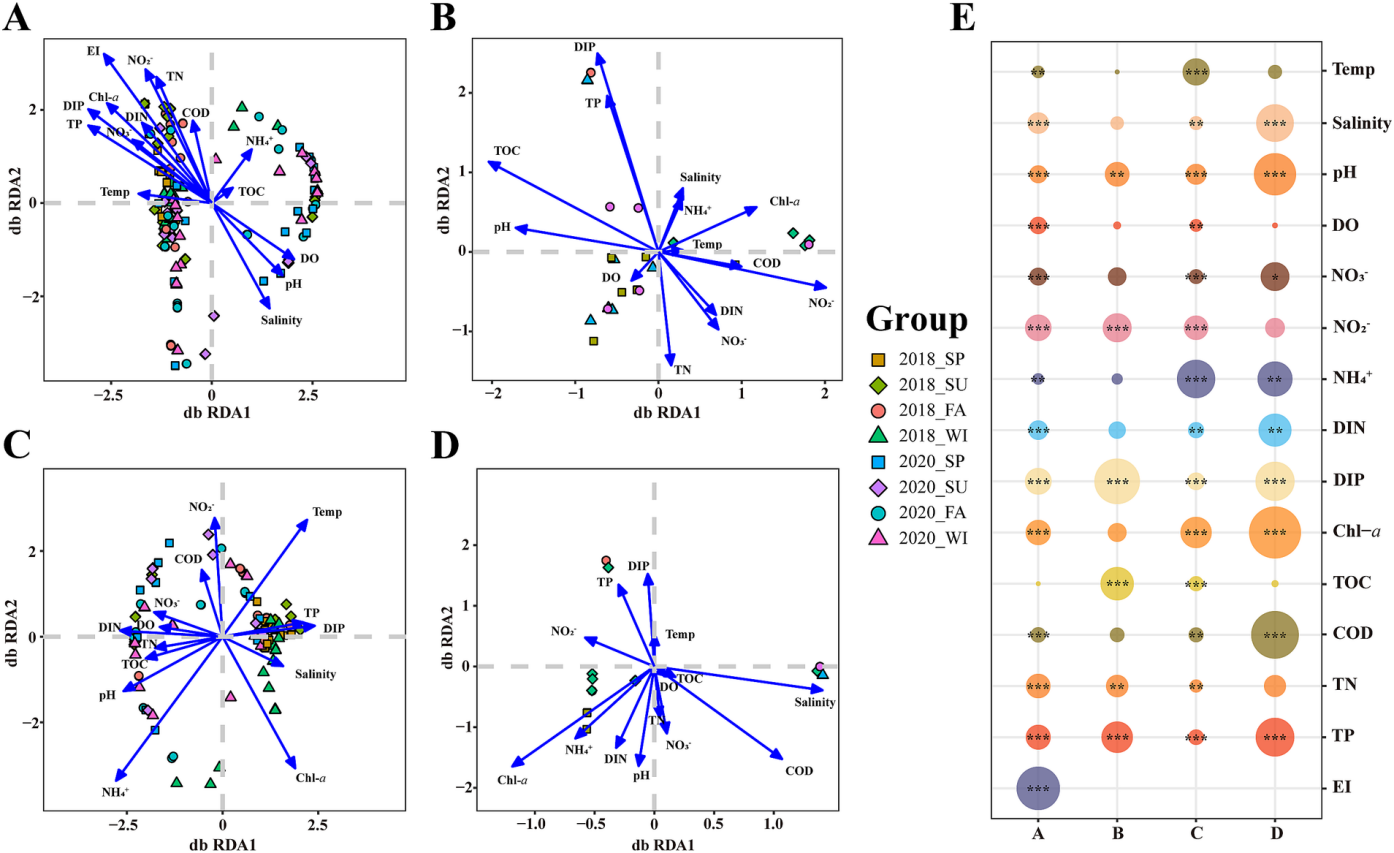
Distance-based redundancy analysis (db-RDA) ordination plots of the relationship between mycoplanktonic communities and environmental factors. **(A)** The total community; **(B)** Oligotrophication; **(C)** Medium eutrophication; **(D)** High eutrophication; **(E)** Relationship between regression coefficient R2 and environmental variables. SP, spring; SU, summer; FA, fall; WI, winter. Temp, temperature; pH, pH; DO, dissolved oxygen; DIN, dissolved inorganic nitrogen; DIP, dissolved inorganic phosphorus; TOC, total organic carbon; COD, chemical oxygen demand; TN, total nitrogen; TP, total phosphorus; EI, eutrophication index. *, *p* < 0.05; **, *p* < 0.01; ***, *p* < 0.001.

We conducted a distance-based redundancy analysis (db-RDA) to clarify the association between the community structure of each group and environmental variables (Figures 3A–D). According to the results, the R^2^ of the environmental factor of db-RDA and the significance *p*-values (Figure 3E). A notable correlation was observed between most environmental variables and the mycoplanktonic community composition. The key environmental factor in the total community was EI (*R*^2^ = 0.1728, *p* < 0.001) (Figures 3A,E). At different eutrophication levels, oligotrophication, medium eutrophication and high eutrophication correspond to DIP (*R*^2^ = 0.2645, *p* < 0.001), NH_4_^+^ (*R*^2^ = 0.1854, *p* < 0.001) and Chl-*a* (R^2^ = 0.4593, *p* < 0.001), respectively (Figures 3B–E).

### 3.3 Community-level change points of mycoplankton in distinct eutrophication levels

**Figure 4:**
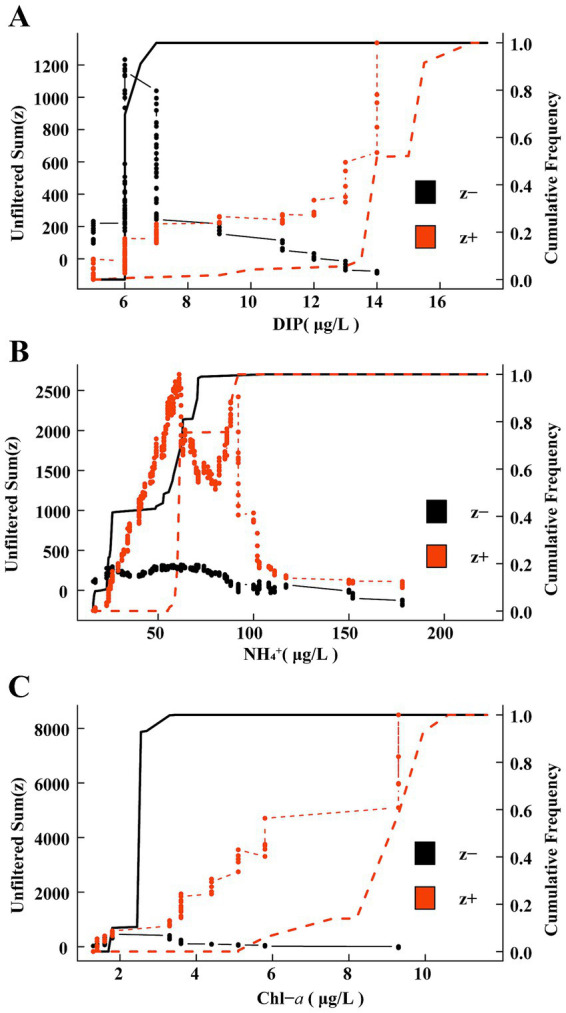
Threshold Indicator Taxa Analysis (TITAN) sums of negative (z-) and positive (z+) of responding species to all candidate change points of key environmental factors. The black solid line and red dotted line depict the cumulative frequency distributions of change points for z- and z + taxa groups, respectively. **(A)** Oligotrophication; **(B)** Medium eutrophication; **(C)** High eutrophication.

The TITAN method was employed to assess the responses of the eutrophication status of the mycoplanktonic community to key environmental factors (Figure 4, Table 1). In oligotrophication group, the most negative (z-) indicator taxa increased rapidly at a low DIP level, and the resulting sum (z-) peaked at 6 μg/L. With increasing DIP level, taxa exhibiting positive responses (z+) increased and peaked at 15.5 μg/L (Figure 4A, Table 1). In the moderate eutrophication group, the majority of the positive (z+) indicator taxa increased sharply at low NH_4_^+^ levels, resulting in a peak sum (z+) of 61.5 μg/L. The response of the negative (z-) indicator taxa to NH_4_^+^ is not significant, which leads to a low sum (z-), and TITAN determines that its peak was 62.5 μg/L (Figure 4B, Table 1). For high eutrophication group, with trends closely resembling the oligotrophication group, the peaks of sum (z+) and sum (z-) were 9.3 μg/L and 2.55 μg/L, respectively (Figure 4C, Table 1). Our findings illustrate that community-level change points for the responses of mycoplankton to oligotrophication and high eutrophication differed between the indicator taxa, demonstrating the most positive (z+) and negative (z-) responses.

**Table 1:** Community-level thresholds of mycoplankton to key environmental factors based on TITAN.

Group	Item	Sum	Env.cp	5%	10%	50%	90%	95%
Oligotrophication	DIP (μg/L)	Sum (z-)	6	6	6	6	6.5	7
		Sum (z+)	15.5	13	14	14	15.5	17
Medium eutrophication	NH_4_^+^ (μg/L)	Sum (z-)	62.5	16.5	23.5	55.5	71	71
		Sum (z+)	61.5	59	59.5	61.5	90	92
High eutrophication	Chl-a (μg/L)	Sum (z-)	2.55	1.8	2.385	2.55	2.55	3.3
		Sum (z+)	9.3	5.8	7.55	9.3	9.95	10.6

### 3.4 Bioindicators of eutrophication in the Dafengjiang River estuary

**Figure 5:**
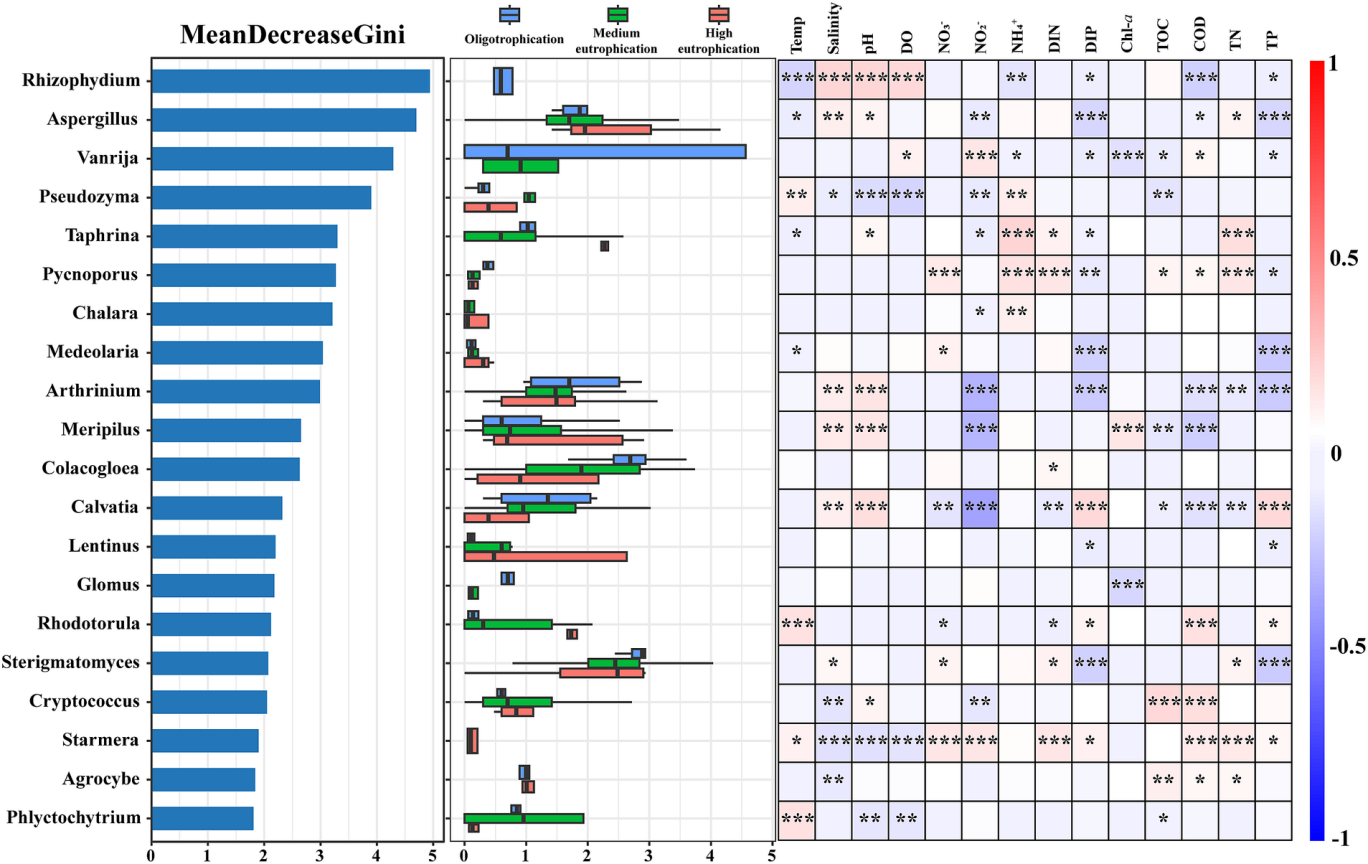
Random Forest classification of the top 20 important genera. Left: Gini index-based analysis of the top 20 taxa, indicating their importance in distinguishing distinct eutrophication levels. Middle: abundance levels of the top 20 genera. Right: Pearson correlations between the relative abundances of the top 20 genera and environmental parameters. Temp, temperature; pH, pH; DO, dissolved oxygen; DIN, dissolved inorganic nitrogen; DIP, dissolved inorganic phosphorus; TOC, total organic carbon; COD, chemical oxygen demand; TN, total nitrogen; TP, total phosphorus. *, *p* < 0.05; **, *p* < 0.01; ***, *p* < 0.001.

The random forest method was applied to determine the most important genera in the samples (Figure 5). The 20 most important indicator genera are displayed based on their maximal Gini values. *Rhizophydium*, *Aspergillus,* and *Vanrija* were the pivotal indicator genera with high Gini values, and their abundances differed in the oligotrophication, medium eutrophication, and high eutrophication groups. Certain mycoplankton genera exhibit diverse adaptive capacities across eutrophication gradients, making them potential indicators of different ecological groups. Spearman’s correlation analysis demonstrated that these 20 genera exhibited significant correlations with multiple environmental parameters (*p* < 0.05) (Figure 5). For instance, *Rhizophydium* exhibited notable positive links with salinity, pH, and DO and notable negative links with COD. *Vanrija* had a significant positive connection with NO_2_^−^ and a significant negative correlation with Chl-*a*, DIP, and TP. *Pseudozym*a was negatively associated with pH, DO, and NO_2_^−^ and positively associated with NH_4_^+^.

## 4 Discussion

Mycoplankton plays a vital role in the cycling of organic matter and nutrients in water bodies (Richards et al., 2012; Li et al., 2019). Given the impact of anthropogenic activities and climate change on subtropical estuarine ecosystems, our understanding of the distribution and community-level change points in mycoplankton remains limited. Our results suggest that eutrophication influences the diversity and community-level change points of mycoplankton in subtropical estuaries. *Rhizophydium*, *Aspergillus,* and *Vanrija* have emerged as bioindicators of eutrophication status in the Dafengjiang River Estuary. Overall, this study clarifies the potential influence of environmental disturbances on mycoplanktonic communities and provides new knowledge for maintaining biodiversity mechanisms in subtropical estuaries.

### 4.1 Environmental fluctuations drive mycoplankton distribution variations in the subtropical estuary

This study explored seasonal and regional variations in the composition and diversity of mycoplankton in subtropical estuarine ecosystems. Our results indicated that the mycoplanktonic communities in the Dafengjiang River Estuary were predominantly Agaricostilbomycetes and Saccharomycetes (Figure 2A). These two classes belong to Basidiomycota and Ascomycota (Souto et al., 2024). Huang et al. (2022) reported that Basidiomycota and Ascomycota classes are abundant in subtropical estuaries. Additionally, Zhang et al. (2021) indicated that Agaricostilbomycetes were commonly present in the Qiantang River Estuary. Lin et al. (2023) reported that Saccharomycetes widely distributed in Shenzhen River Estuary. These observations demonstrate the widespread distribution of Agaricostilbomycetes and Saccharomycetes in estuarine ecosystems, which may be attributed to the combined effects of unique climatic conditions and anthropogenic influences. At the class level, the composition of the mycoplanktonic community varied seasonally and regionally (Figure 2A and Supplementary Figure S1). The Dafengjiang River Estuary is a typical subtropical eutrophic estuary that drains directly into Beibu Gulf, forming an estuary-bay multi-ecosystem that is exposed to various pressures (Lai et al., 2014). This observation may explain the seasonal and regional dominant class shifts in the Dafengjiang River Estuary. Furthermore, certain mycoplankton were maintained at a lower abundance level, suggesting that they are not extensively distributed in the estuary environment of the Dafengjiang River and may fulfill a particular ecological function (Xue et al., 2018).

The alpha diversity of the mycoplankton exhibited distinct seasonal and regional changes in each group (Figure 2B and Supplementary Figure S2). Consistent with prior studies, Husanovic reported that seasonal changes affect fungal community diversity (Husanovic, 2021). Maximum values were observed in spring 2020, whereas minimum values were observed in summer 2020 (Figure 2B). These reduced levels may be attributed to adverse summer conditions, such as high temperatures (Wang et al., 2021), which inhibit mycoplanktonic development. Notably, we found that the average water temperatures in the summer of 2020 were higher than those in the other seasons, which explains why the lowest level of alpha diversity was observed in the summer of 2020 (Wang et al., 2021). Additionally, the alpha diversity of mycoplankton varied seasonally across the different areas (Supplementary Figure S2). This may result from differences in the extent of seasonal variations in the physicochemical properties of water across regions. Nutrient status and resource availability play critical roles in shaping fungal diversity (Sen et al., 2022; Lobo et al., 2024), suggesting the potential impact of the environment on mycoplanktonic community diversity in subtropical estuaries. Beta diversity analysis indicated significant seasonal variations in mycoplanktonic community structure (Supplementary Figure S3), demonstrating the high variability in fungal communities under environmental heterogeneity (Wang et al., 2019). Overall, the mycoplanktonic community structure displays a specific distribution and variation with environmental fluctuations in subtropical estuaries.

### 4.2 Eutrophication affects mycoplanktonic community and its thresholds

The db-RDA analysis demonstrated that eutrophication status as the principal factor shaping mycoplanktonic community variation in the Dafengjiang River Estuary (Figure 3). In accord with recent studies, there was a strong correlation between mycoplankton diversity and eutrophication in coastal ecosystems (Wang et al., 2018; Wang et al., 2019; Zhao et al., 2023). Anthropogenic activities, including agricultural fertilization, industrial effluent discharge, and domestic sewage release input excessive nitrogen and phosphorus into the coastal environment. This process changes the physicochemical properties of coastal water bodies, and subsequently affects the structure and diversity of mycoplanktonic communities (Meybeck, 2003). Geng et al. (2022) found that eutrophication strongly influences the diversity of aquatic fungal communities. This effect is caused by multiple pathways, including habitat heterogeneity, environmental filtering, and the regulation of interspecies interactions (Geng et al., 2022). In addition, we studied the principal environmental factors along the eutrophication gradients, which were identified as DIP, NH_4_^+^, and Chl-*a* (Figure 3). This is consistent with previous studies, further validating their roles in shaping community dynamics. For instance, phosphate is the primary limiting nutrient in most estuaries in China (Harrison et al., 1990; Pinckney et al., 2001). The distribution of mycoplanktonic communities was significantly influenced by phosphate, which is consistent with observations in the coastal waters of the Bohai Sea (Wang et al., 2018). Duarte et al. (2009) reported that fungal communities were significantly correlated with NH_4_^+^ concentrations in eutrophic water bodies. Gao et al. (2024) also found that the environmental changes in nitrogen and phosphorus levels can significantly impact fungal community structure and species composition. Zhao et al. (2023) suggested that Chl-*a* is an important factor influencing mycoplanktonic communities in highly eutrophic estuaries. An increase in Chl-*a* concentration indicates an increase in the primary productivity of the overall community, which may lead to changes in the ecological niche of mycoplankton. In summary, mycoplanktonic communities across different eutrophication levels were driven by distinct environmental factors, suggesting that eutrophication affects the assembly of mycoplanktonic communities.

Clarifying the thresholds for distinct eutrophication statuses is essential for assessing the relationships between biological populations and the environment as well as for ecological conservation. In this study, we used TITAN to determine the thresholds for mycoplanktonic communities (Figure 4, Table 1). Most of the indicator groups exhibited strong indicator states for key environmental parameters, indicating the effective indicative characteristics of mycoplankton. Threshold responses of mycoplanktonic community structure to DIP (6–15.5 μg/L), NH_4_^+^ (61.5–62.5 μg/L) and Chl-*a* (2.55–9.3 μg/L) along the eutrophication gradients (Figure 4, Table 1). This demonstrates that exceeding this range for each trophic status may cause instability in the community and even trigger ecological imbalance. Additionally, this study provides strong evidence that eutrophication influences the diversity and community-level change points of mycoplankton, indicating that mycoplanktonic communities can sustain a relatively stable ecological balance along the eutrophication gradients. Mycoplankton are crucial for nutrient cycle and energy flow, exhibit a strong correlation with environmental factors, and can swiftly respond to aquatic environment shifts (Hyde et al., 1998). In the oligotrophication zone, the concentration of phosphorus can influence parasitism (Van Donk and Bruning, 1995) and participation in phosphorus cycling (Peng et al., 2024) by fungi, which may trigger resource competition or niche changes, leading to mycoplanktonic community sensitivity to phosphorus concentration. In the medium eutrophication group, the DIP threshold of the mycoplanktonic community shifted to the NH_4_^+^ threshold. Fungi exhibit high plasticity in the C: N ratio of their cellular biomass (Danger and Chauvet, 2013; Danger et al., 2016), which may allow them to survive in ammonium-limited environments. However, high ammonium concentrations in water most likely have negative effects on mycoplanktonic communities (Duarte et al., 2009). In high eutrophication zones, the increase in phytoplankton biomass stimulates mycoplankton and their parasitic relationships and participates in material transfer (Miki et al., 2011). Overall, this study established an integrated analytical framework employing TITAN to clarify the community-level change points of mycoplankton, and offers theoretical references for environmental monitoring.

### 4.3 Mycoplankton indicator genera in the Dafengjiang River estuary

Mycoplankton demonstrate rapid responsiveness to environmental fluctuations in aquatic environments, making them reliable bioindicators for detecting and assessing environmental disturbances (Hyde et al., 1998; Sole et al., 2008; Parmar et al., 2016). In this study, *Rhizophydium*, *Aspergillus,* and *Vanrija* emerged as crucial indicator genera and were sensitive to changes in eutrophication levels within the Dafengjiang River Estuary (Figure 5). The ability of these mycoplankton to indicate environmental changes may be attributed to their distinct features. For example, the genus *Rhizophydium* was negatively connected with the COD (*p* < 0.001). *Rhizophydium* has the potential to suppress the occurrence of cyanobacterial blooms and contribute to water quality (e.g., COD, NH_4_^+^ and DIP) regulation through its ecological functions (Lai et al., 2021). *Vanrija* is a genus of certain species of *Candida* (Moore, 1980), the phosphorus concentration in the environment is regulated by the Pho84 protein, which affects its ability to invade the host (Acosta-Zaldivar et al., 2024). Multiple correlations were observed between the indicated genera and nitrogen and phosphorus (Figure 5). This finding is consistent with prior findings indicating that the access of fungi to nitrogen and phosphorus in the environment varies depending on their lifestyle (Root, 1967; Polme et al., 2020). Therefore, these indicator genera are sensitive to eutrophication levels and may be useful for assessing the trophic status in the Dafengjiang River Estuary.

## 5 Conclusion

This study elucidated the structure and community-level change points of the mycoplanktonic community in the Dafengjiang River Estuary. Agaricostilbomycetes and Saccharomycetes were the dominant classes. Eutrophication level was a key driver influencing variation in the mycoplanktonic community. DIP, NH_4_^+^, and Chl-*a* were principal environmental drivers along the eutrophication gradients and their nutrient criteria were 6–15.5, 61.5–62.5, and 2.55–9.3 μg/L, respectively. *Rhizophydium*, *Aspergillus,* and *Vanrija* can serve as indicator genera to reflect changes in the eutrophication status in the studied regions. This study elucidated the environmental thresholds of mycoplanktonic communities in subtropical estuaries and established a scientific foundation for comprehending the nutrient criteria for mycoplanktonic community stability in response to anthropogenic and climatic stressors in subtropical estuaries.

## Data Availability

The original contributions presented in the study are publicly available. This data can be found here: https://www.ncbi.nlm.nih.gov/genbank/, accession numbers: PRJNA1044415 and PRJNA866330.
