# Supplementary material for: Eutrophication influences diversity and community-level change points of mycoplankton in subtropical estuaries
Source: Front Microbiol. 2025 Jun 27;16:1620942. doi: 10.3389/fmicb.2025.1620942 (PMC12245875; doi:10.3389/fmicb.2025.1620942)
Supplement: Supplementary file 1 [file Data_Sheet_1.docx]

Supplementary Material


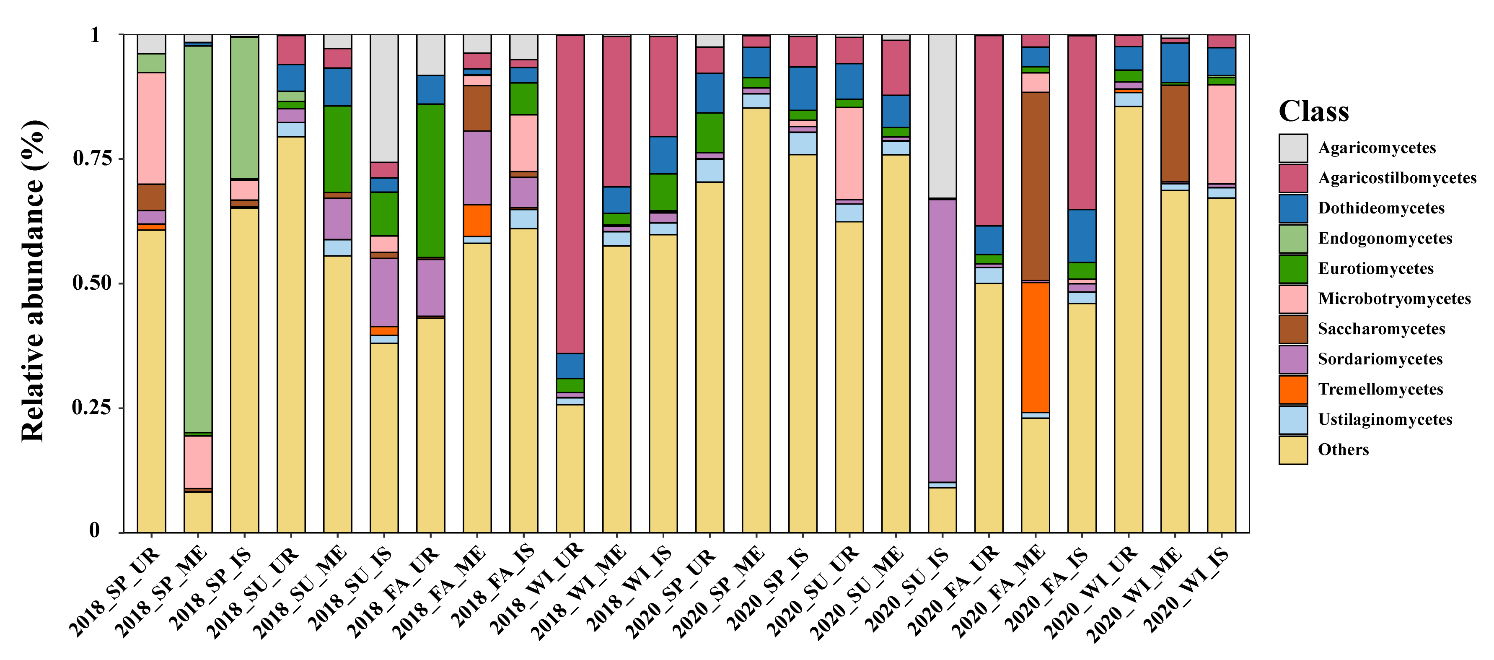


**Supplementary Figure 1.** Mycoplanktonic community composition at different years (2018 and 2020) and seasons in distinct regions of Dafengjiang River Estuary were evaluated based on relative abundance of different taxa at the class level. SP, spring; SU, summer; FA, fall; WI, winter. UR, upper reaches; ME, middle estuary; IS, inner shelf.


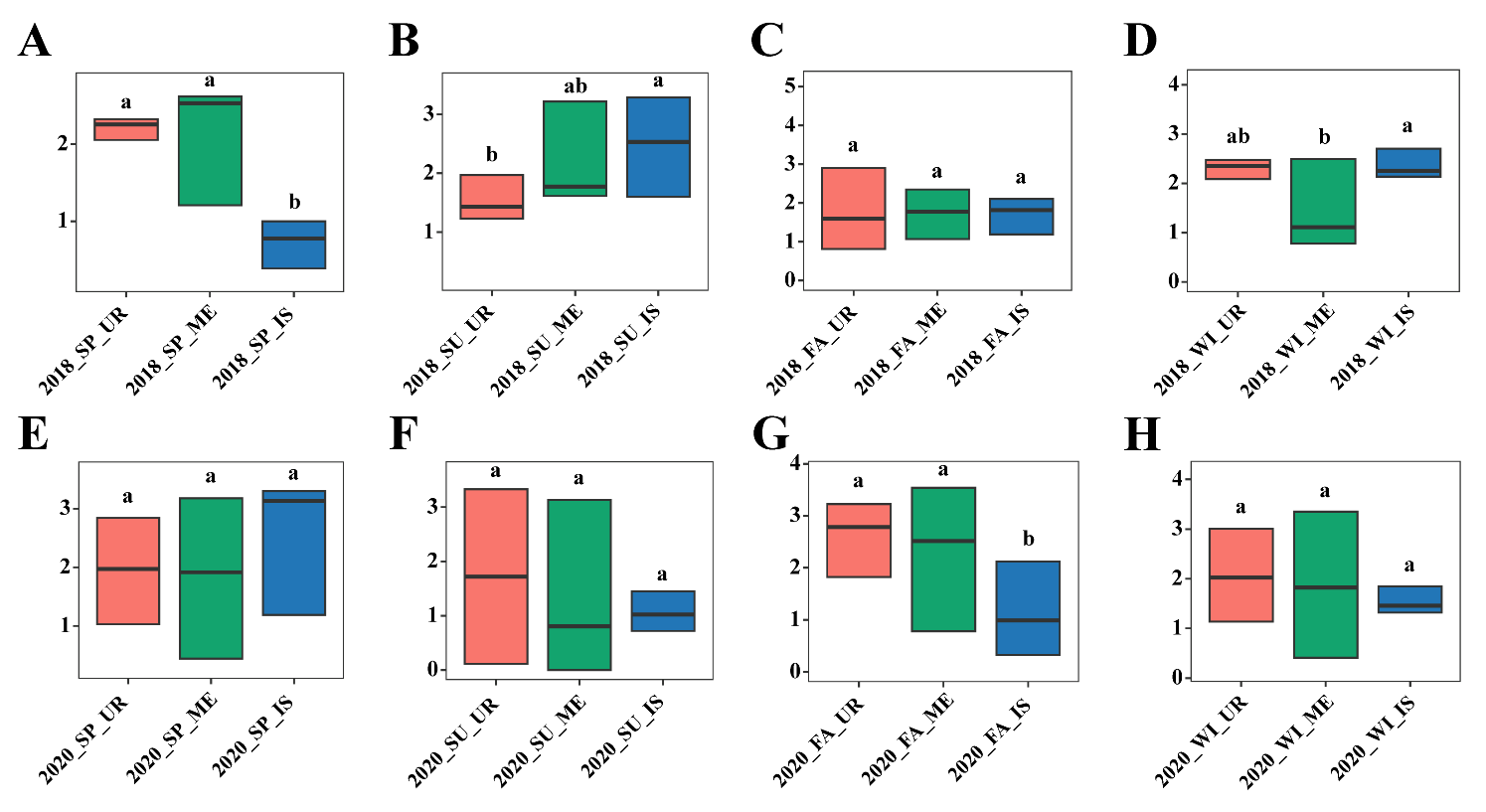


**Supplementary Figure 2.** The alpha diversity (Shannon) at different years (2018 and 2020) and seasons in distinct regions of Dafengjiang River Estuary. The alpha diversity shown by boxplot. SP, spring; SU, summer; FA, fall; WI, winter. UR, upper reaches; ME, middle estuary; IS, inner shelf.


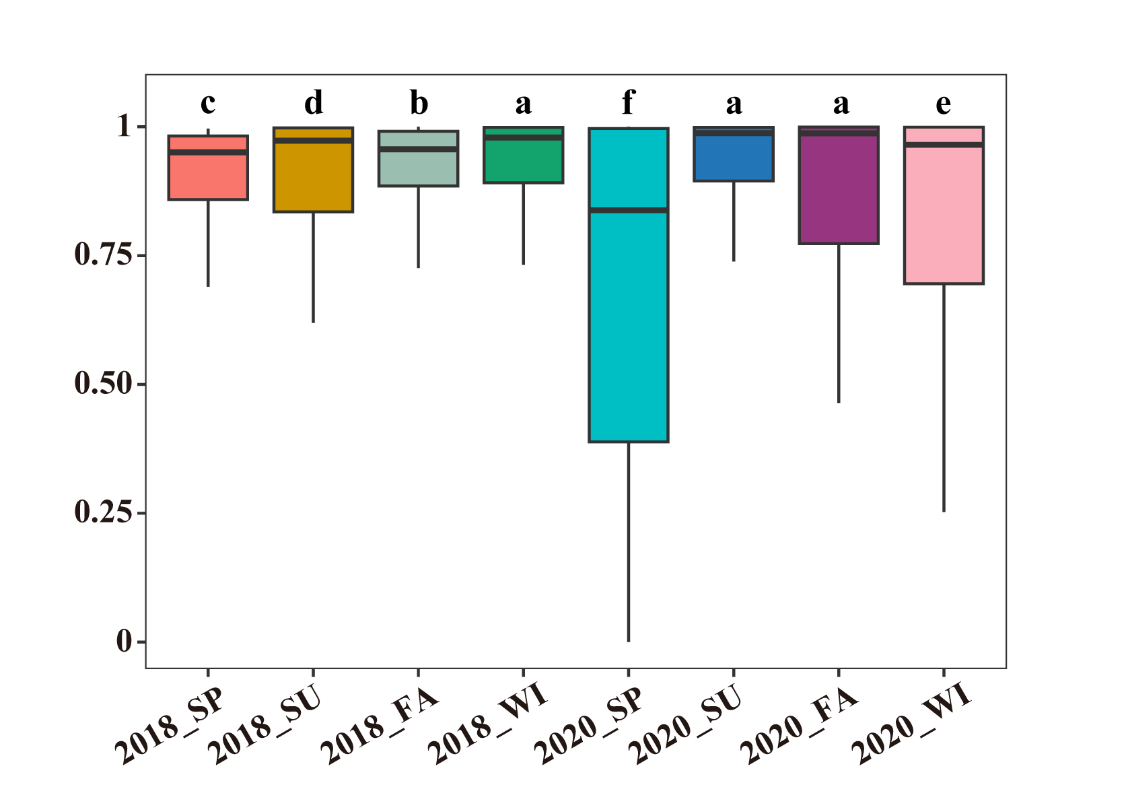


**Supplementary Figure 3.** The beta diversity at different years (2018 and 2020) and seasons. The beta diversity among samples estimated based on a Bray-Curtis distance matrix and shown by boxplot. SP, spring; SU, summer; FA, fall; WI, winter.
